# An autochthonous case of severe *tick-borne encephalitis* virus associated meningoencephalitis in France: Is there a place for polyvalent intravenous immunoglobulins?

**DOI:** 10.1016/j.idcr.2025.e02213

**Published:** 2025-03-31

**Authors:** Paul Dalmas, Elsa Kaphan, Coline Mortier, Margaux Froidefond, Barbara Doudier, Laeticia Ninove, Antoine Nougairede, Guillaume André Durand, Jean-Christophe Lagier, Nadim Cassir

**Affiliations:** a IHU Méditerranée Infection, AP-HM, Marseille, France; b Division of Internal Medicine and Clinical Immunology, Hôpital Conception, APHM, Marseille, France; c Unité des Virus Émergents (UVE: Aix-Marseille Univ, Università di Corsica, IRD 190, Inserm 1207, IRBA), France; d National Reference Center for Arboviruses, Inserm-IRBA, Marseille, France; e Aix-Marseille Université, IRD, MEPHI, Marseille, France

**Keywords:** Tick-borne encephalitis, Tick-borne encephalitis virus, Intravenous immunoglobulins

## Abstract

Tick-borne encephalitis virus (TBEV) is a common cause of viral encephalitis in parts of Central and Eastern Europe, with a recent resurgence of cases and geographical expansion. Active immunization results in a high rate of seroconversion and is the most effective measure to reduce the incidence of tick-borne encephalitis (TBE). In France, an endemic country, vaccination is recommended only for travelers staying in rural or forested areas in endemic regions. Polyvalent intravenous immunoglobulin (IVIG) is sometimes used as rescue treatment of viral encephalitis. However, few cases of TBEV meningoencephalitis treated with polyvalent IVIG have been described. We report here a case of autochthonous TBEV meningoencephalitis in a French patient with cranial nerve involvement that was treated with IVIG and discuss the possible mechanisms of action.

## Introduction

Tick-borne encephalitis (TBE) is a vaccine-preventable disease caused by tick-borne encephalitis virus (TBEV), a member of the genus *Orthoflavivirus*. TBEV is transmitted to humans primarily by the bite of an *Ixodes* spp. tick and, less commonly, by consumption of unpasteurised dairy products [1]. Endemic areas are northeastern Asia and Europe, including Russia, the Baltic States, Scandinavia, and Eastern and Central Europe [2]. In Europe, TBE-Eu is an emerging seasonal (summer) infection with increasing numbers of cases and geographical spread. In 2021, 2949 confirmed cases of TBE were reported by European countries to the European Centre for Disease Prevention and Control (ECDC) [3].

Typically, after 4–28 days of incubation period, TBE presents as a biphasic disease, with a short flu-like illness followed by meningitis, meningoencephalitis, or meningomyelitis [4]. Cranial nerves involvement varies between 1.1 % and 8.8 % of reported cases [4]. In a study of 11 patients with facial nerve palsy associated with TBEV, mean delay of development was 12 days after the beginning of the meningoencephalitis phase [5]. Up to 20 % of patients suffer from long-term sequelae after recovery, including cognitive and movement impairments [6]. Recently the European cohort EU-TICK-BO reported a low mortality-rate of 0.9 %, but a high persisting neurological impairment in 59 % of the surviving patients [4].

Polyvalent intravenous immunoglobulins (IVIG) have been widely used for treatment of autoimmune diseases such as primary immune thrombocytopenia or Guillain-Barré syndrome [7]. A recent systematic literature review by Wagner et al. reported 39 cases of viral encephalitis treated with IVIG with heterogeneous results concerning efficacy [8].

To the best of our knowledge, few cases of TBEV meningoencephalitis treated with polyvalent IVIG have been described so far [9], [10]. We report here a case of autochthonous TBEV meningoencephalitis in a French patient with cranial nerve involvement that was treated with polyvalent IVIG.

## Case report

In June 2023, a man aged about fifty years was referred to the emergency department with severe headache and fever. He had a history of chronic migraine, well controlled with non-steroidal anti-inflammatory drugs. He reported a trip to Mens in the Isère department, South-east France, 2 weeks ago. He went hiking and ate unpasteurized fresh goat's cheese. He didn't notice any tick bite, but mentioned a fever of a few days' duration with a favorable spontaneous course. Physical examination revealed neck stiffness and the rest of the neurological examination was normal. Laboratory tests revealed inflammation with leukocytosis (10 × 10⁹/L; normal range: 4–10 × 10⁹/L), elevated polynuclear neutrophils (9.24 × 10⁹/L; normal range 1.5–7 × 10⁹/L), monocytosis (1.13 × 10⁹/L; normal range 0.2–0.8 × 10⁹/L), lymphopenia (0.89 × 10⁹/L; normal range: 1.4–4 × 10⁹/L) and C-reactive protein elevation (20 mg/L; normal range <5 mg/L). Liver enzymes and electrolytes were normal. A cerebral computed tomography scan was normal. Lumbar puncture was performed and cerebrospinal fluid (CSF) analysis showed pleocytosis with 207 white blood cells per milliliter with 55 % lymphocytes, 35 % polynuclear neutrophils and 20 % monocytes. Glycorrhachia was 3.94 mmol/L (normal range 2.20–3.90 mmol/L), proteinorrhachia was elevated at 0.96 g/L (normal range 0.15–0.45 g/L). Direct examination and molecular biology tests including the Biofire® FilmArray® panel (Biomérieux, Lyon, France) for meningitidis/encephalitis (multiplex real-time polymerase chain reaction (PCR) targeting Enterovirus, HSV, VZV, *Escherichia coli, Hemophilus influenzae, Listeria monocytogenes, Neisseria meningitidis, Streptococcus agalactiae, Streptococcus pneumoniae*, CMV, Parechovirus) were negative. Reverse transcription PCR (RT-PCR) for TBEV in the CSF was negative [11]. He was started on probabilistic intravenous treatment with ceftriaxone 100 mg/kg per day and amoxicillin 200 mg/kg per day. In addition, RT-PCR West Nile [12] and Toscana [13] viruses in the CSF were negative. CSF serology was also positive in IgM (ratio: 3.8; positive > 3; negative < 2.5). Serum serologies were negative for *Borrelia burgdorferi* (chemiluminescence immunoassay, Diasorin), *Brucella melitensis* (Rose Bengal test, Vircell), *Francisella tularensis* (indirect immunofluorescence (IFI), in-house test), *Coxiella burnetiid* (IFI, in house test), *Rickettsia conorii* (IFI, in house test), *Bartonella henselae* (IFI, in house test), *Anaplasma phagocytophilum* (IFI, in house test) as well as dengue (ELISA, Euroimmun) and Zika virus (ELISA, Euroimmun). CSF serology for *Borrelia Burgdoferi* was negative (western-blot assay, Mikrogen Diagnostik). Antibiotics were stopped. After two weeks of progressive improvement of the meningitis, the patient developed new severe headaches and dysphagia. Physical examination revealed rapidly progressive damage to the cranial nerves, over a few days, including bilateral facial palsy (7th cranial nerve) of grade VI on the right and IV on the left according to the House-Brackmann classification, left corneal hypoesthesia (5th cranial nerve) and abolition of the nausea reflex. Cerebral magnetic resonance imaging with contrast was normal. It was decided to start polyvalent IVIG at 0.4 g/kg per day for five days (Clairyg®, LFB Biomédicaments, Paris-Saclay, France). Within a few days, the dysphagia rapidly resolved and the bilateral facial paralysis partially improved. Follow-up at 1 month showed discreet left orbital palsy and corneal hypoesthesia with no swallowing difficulties. Follow-up at 3 months showed persistent corneal hypoesthesia but no swallowing difficulties or facial paralysis. Follow-up at 6 months showed no more involvement of cranial nerves.

## Discussion

We report here a case of autochthonous TBEV meningoencephalitis with cranial nerve involvement who, after treatment with polyvalent IVIG, rapidly showed signs of improvement. This case comes at a time of alert, with a TBE emergence in Europe, being partly explained by increased exposure to ticks with geographic expansion of the Ixodes tick vector (*Ixodes ricinus* in France) due to climate change, and, in some areas, declining vaccination rates [14]. Since it became a mandatory notifiable disease in France, public health authorities have recorded 71 cases of TBE between May 2021 and May 2023 [15]. The sex ratio was 1.7 and cases ranged in age from 7 to 80 years (median 48 years). Of these cases, 61 (86 %) were autochthonous and 10 (14 %) were imported from other countries. Most of them developed central nervous system symptoms: 26 (37 %) cases of meningitis, 27 (38 %) cases of encephalitis, 9 (13 %) cases of meningoencephalitis and meningoencephalitis and 2 (3 %) cases of encephalomyelitis. No deaths have been reported, although most (94 %) were admitted to hospital. Sixty-nine cases (97 %) were diagnosed by serology: 17 had positive IgM serology in the CSF and 64 had positive IgM and IgG serologies in the serum. Seroconversion was detected in two cases. One case had a positive RT-PCR on the CSF and one case had a positive RT-PCR in serum [15]. In our case, while PCR for TBEV in the CSF was negative, both serum and CSF TBEV serologies were positive with confirmed seroconversion measured before administration of IVIG. Moreover, among the recorded 71 cases of TBE recorded in France between May 2021 and May 2023, 36 (51 %) reported a tick bite within a timeframe compatible with their date of onset of the signs. Eighteen (25 %) cases reported consumption of raw milk or raw milk dairy products [15]. In our case, the most probable route of infection is eating unpasteurized fresh goat's cheese. Physicians should therefore be aware of the presence of TBEV in France and inform their patients planning to do outdoor activities in rural or forested areas that they should inspect their whole body daily for ticks to remove them quickly, and avoid consuming unpasteurized dairy products.

**Fig. 1:**
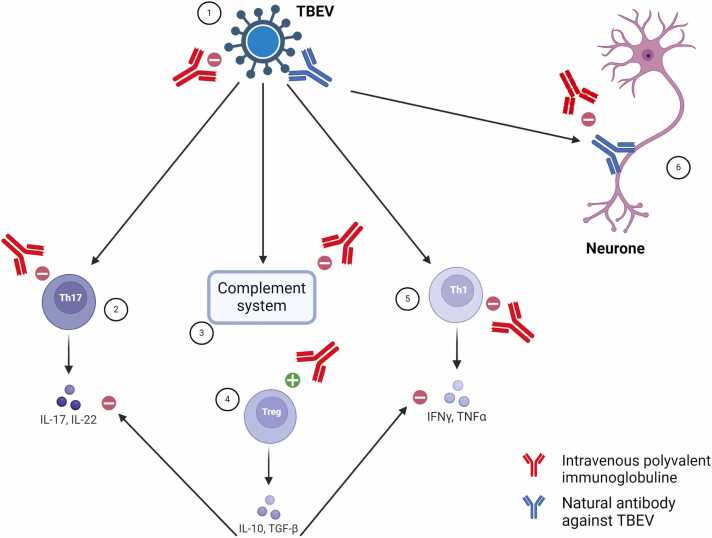
Putative mechanisms of action of polyvalent intravenous immunoglobulins in tick-borne encephalitis virus associated meningoencephalitis. 1. direct inhibition by anti-TBEV specific antibody. 2. inhibition of the Th17 response. 3. inhibition of the complement system. 4. activation of Treg that lead to suppression of Th1 and Th17 responses. 5. inhibition of Th1 response. 6. inhibition of natural antibody against TBEV that cross reacts and attacks neuronal tissue. TBEV = tick-borne encephalitis virus.

Active immunization results in a high rate of seroconversion and is the most effective measure of decreasing the incidence of TBE as there is no specific antiviral agent available against TBEV [14]. In France, vaccination against TBEV is recommended for travelers staying in rural or wooded areas in endemic regions (Central, Eastern, and Northern Europe; Northern Central Asia; Northern China; and Northern Japan), from spring to autumn, and was therefore not recommended for our patient [16]. It has previously been shown that TBEV-specific antibody content of IVIG had a highly neutralizing effect in cell culture and correlated with protection from disease in mice [17]. From a mechanistic point of view IVIG can be used for their immunomodulatory properties but also for their direct antiviral effects (Fig. 1). Notably, the seroprevalence of anti-TBEV antibodies in immunoglobulin replacement products can differ widely, mainly due to the endemic nature of the disease and varying vaccination coverage in donor populations [18], [19]. For example, in a recent Swedish case of severe TBE in a patient with X-linked agammaglobulinemia, even though the patient received subcutaneous IgG replacement therapy (Hizentra, CSL Behring), no neutralizing antibodies against TBEV were detected in the patient’s serum [20]. Treatment with additional IVIG was ineffective while the patient improved after receiving a plasma unit containing specific TBEV antibodies (IgG). In the present case, the efficacy of IVIG is more likely secondary to its immunomodulatory properties because of the poor prevalence of TBEV antibody in French donors.

In fact, cranial nerve involvement in TBE occurs with an average delay of twelve days after the onset of the meningoencephalitis phase [5]. Numerous data suggest that the pathogenesis of TBE is characterised by a progressive decrease in TBEV viral load and a progressive overactivation of the immune system leading to neurological damage [1]. This delayed involvement of cranial nerves two weeks after the onset of meningitis leads us to suspect an immune-mediated mechanism and therefore to consider immunomodulatory treatment. The European Academy of Neurology guidelines for the management of TBE don't recommend immunomodulatory therapies and suggest symptomatic therapy, which we have already started [21]. However, as acute cranial nerve palsy is a peripheral nerve disorder, we decided to use IVIG in the same way as in Guillain-Barré syndrome [7]. This decision was based on a previous description of IVIG in TBE by Kleiter et al. They used IVIG in a 54-year-old man who developed peripheral nerve involvement with persistent dysautonomia, facial palsy and upper limb paresis 10 days after the onset of the meningoencephalitis phase of TBE [9]. As in our patient, IgG anti-TBE levels rise sharply from 403 to 1151 between day 4 and day 15, shortly after the onset of cranial involvement (ELISA, Enzygnost® Anti-TBE Virus (IgG, IgM); Dade Behring, Marburg, Germany).

To better understand how IVIG prevents tissue damage in TBE, we will describe the four different pathways that cause neuroinflammation in TBE and the ways in which IVIG inhibits them. Indeed, neurological lesions in TBE appear to be mediated by inflammation rather than direct viral action [1]. First, IVIG inhibits T-helper (Th)17 immunity. Indeed, Th17 responses have been shown to be involved in TBE in facilitating neutrophilic infiltration of the central nervous system, particularly via interleukin 8 and C-X-C motif ligand 1, but also interleukin 17A and 17F [22]. Interaction between the fragment antigen-binding region (Fab) of IVIG and Th17 cells reduces the expression of retinoic acid-related orphan receptor C and signal transducer and activator of transcription 3 in Th17 cells, which impairs their differentiation and proliferation as well as the production of Th17-related cytokines (interleukins 17A, 17F and 22) [23]. IVIG also inhibits TH17 by expanding T regulatory (Treg) lymphocytes, which dampen the TH17 response [24]. Secondly, IVIG inhibits Th1 immunity. In fact, CSF analysis during the neurological phase shows overexpression of cytokines associated with the Th 1 adaptive immune system [25] compared to peripheral blood, suggesting involvement of these pathways in the pathogenesis of TBE. In particular, Th1 is associated with the severity of TBE [24]. IVIG inhibits the Th1 pathway by expanding Treg lymphocytes [24]. Thirdly, IVIG inhibits the complement system. Indeed, the classical pathway of the complement system has been shown to be increased in the CSF of TBE patients [25]. This activation could lead to the formation of the membrane attack complex that drives neuronal lysis [25]. IVIG inhibits complement by intercepting C3a and C5a with its Fab domain [7]. Fourthly, IVIG inhibits antibody responses.

Indeed, it is possible that a humoral response directed against TBEV may also be directed against a component of the nerve, due to molecular mimicry between the virus and the nerve, leading to the development of autoantibodies. This phenomenon has been described, for example, in Guillain-Barré syndrome secondary to *Campylobacter jejuni* infection [26]. IVIG could intercept TBEV-driven autoantibodies by complexing with them and directing them for elimination, as is the case in several autoimmune diseases [7].

IVIG may be considered as a potential rescue therapy for TBE with late and severe manifestations suggesting immune overactivation. However, we could not rule out that the favorable response was due to the natural course of the disease. Further work is needed to better understand whether the mechanism of action is through modulation of the exaggerated immune response or through the neutralizing effect of specific TBEV antibodies, or both.

## Ethical approval

None.

## Consent

Written informed consent has been signed by the patient

## Funding sources

This research did not receive any specific grant from funding agencies in the public, commercial, or not-for-profit sectors.

## CRediT authorship contribution statement

**Antoine Nougairede:** Writing – review & editing, Validation, Supervision, Resources, Methodology, Investigation, Formal analysis. **Coline Mortier:** Data curation. **Margaux Froidefond:** Data curation. **Barbara Doudier:** Data curation. **Laetitia Ninove:** Visualization, Investigation, Data curation. **Nadim Cassir:** Writing – original draft, Supervision, Methodology, Investigation, Formal analysis, Data curation, Conceptualization. **Paul Dalmas:** Writing – original draft, Resources, Investigation. **Elsa Kaphan:** Writing – review & editing, Data curation. **Guillaume Durand:** Supervision, Resources, Methodology, Investigation, Data curation. **Jean-Christophe Lagier:** Visualization, Validation, Supervision, Resources, Investigation, Conceptualization.

## Declaration of Competing Interest

The authors declare that they have no known competing financial interests or personal relationships that could have appeared to influence the work reported in this paper.
